# Emerging Antimicrobial Resistance in *Klebsiella pneumoniae*: A Molecular and Antibiogram Insight From the Beef Value Chain in Bangladesh

**DOI:** 10.1002/mbo3.70263

**Published:** 2026-03-25

**Authors:** Md Sodor Uddin, Fahmida Jahan Fahim, Sohel Rana, Abdullah Al Kafi, Jobaida Khanam, Md Nazim Uddin, Md Masudur Rahman, Monira Noor, Md Mukter Hossain, Md Mahfujur Rahman, Md Bashir Uddin, Ferdaus Mohd Altaf Hossain

**Affiliations:** ^1^ Department of Dairy Science, Faculty of Veterinary, Animal and Biomedical Sciences Sylhet Agricultural University Sylhet Bangladesh; ^2^ Department of Livestock Production & Management, Faculty of Veterinary, Animal and Biomedical Sciences Sylhet Agricultural University Sylhet Bangladesh; ^3^ Department of Pathology, Faculty of Veterinary, Animal and Biomedical Sciences Sylhet Agricultural University Sylhet Bangladesh; ^4^ Department of Medicine, Faculty of Veterinary, Animal and Biomedical Sciences Sylhet Agricultural University Sylhet Bangladesh

**Keywords:** antimicrobial resistance, beef, biofilm, *Klebsiella pneumoniae*, public health

## Abstract

*Klebsiella pneumoniae* is an opportunistic pathogen linked to rising antimicrobial resistance (AMR) globally. To assess the antimicrobial resistance pattern and biofilm‐forming ability of *K. pneumoniae*, a total of 240 samples were collected from slaughterhouses, open butcher shops, wet market selling points, and high‐grade, medium‐grade, and poor‐grade restaurants in Dhaka City Corporation (DCC) and Gazipur City Corporation (GCC). Among the samples, 132 (55%) samples were positive for *K. pneumoniae*, with the highest prevalence (60%) in raw beef from GCC. The antibiogram profile depicted diverse resistance patterns with the highest resistance pattern to ampicillin (100%), amoxicillin (100%), and cefoxitin (87.12%). All isolates exhibited a multiple antibiotic resistance index (MARI) value greater than 0.2, indicating contamination from high‐risk sources. The antimicrobial resistance encoding genes were *bla*_*BIC*_ and *bla*_*IMP*,_ were 54% and 36%, respectively. Phenotypic characterization, utilizing Congo red agar and microtiter plate tests, identified 25 out of 132 (19%) isolates as biofilm producers, with 7 (28%) classified as strong producers, and 3 (12%) and 15 (60%) as intermediate and weak producers, respectively. This study addresses the alarming emergence of antimicrobial resistance within the beef value chain in Bangladesh, posing an alarming threat to food safety and public health associated with biofilm‐producing foodborne pathogens, underscoring the necessity for improved hygiene practices to mitigate the public health risk posed by these pathogens.

## Graphical abstract

A total of 132 Multidrug‐resistant *Klebsiella pneumoniae* was detected across the beef value chain in Bangladesh, including slaughterhouses, wet markets, and ready‐to‐eat beef outlets. Upon cultural traits, PCR, 16S rRNA sequencing, antibiogram analysis, and ARG detection revealed carbapenem resistance genes (*bla*IMP and *bla*BIC), highlighting foodborne transmission risks within the One Health interface in Bangladesh.

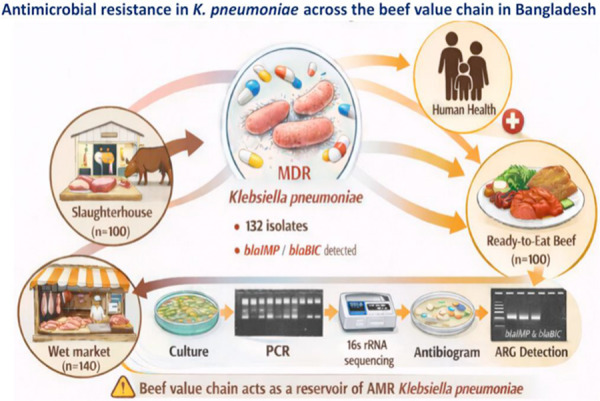

## 1 Introduction

The safety of the beef value chain is a major concern in Bangladesh, due to the rapidly expanding livestock sector, where meat consumption is a significant constituent of the diet (Abdul et al. 2002; Islam et al. 2018). The presence of foodborne pathogens, such as *Klebsiella pneumoniae*, causes severe infections and increases resistance to antibiotics, which poses a remarkable public health threat (Paczosa and Mecsas 2016; Hartantyo et al. 2020). In the recent decade, the increasing antibiotic resistance among different Gram‐negative and Gram‐positive bacteria has posed a serious challenge to healthcare systems globally (Afrough et al. 2012; Jomehzadeh et al. 2022; Abbasi Montazeri et al. 2020; Raouf et al. 2022). *K. pneumoniae* is a significant opportunistic pathogen under the *Enterobacteriaceae* family, which commonly causes infections in the respiratory system, urinary system, and bloodstream in humans (Paczosa and Mecsas 2016; Fahim et al. 2025). In recent years, its prevalence in raw and processed meat and meat products has posed a considerable public health threat due to its role in foodborne illnesses and antimicrobial resistance (AMR) transmission (Conceição et al. 2023; Rana et al.).

The beef value chain operates in a complex channel in Bangladesh, including established slaughterhouses, meat processing units, wet markets, and food vendors. Lack of hygiene practices, suboptimal cold‐chain maintenance, and potential cross‐contamination at various points in the marketing channel significantly increase the risk of microbial contamination (Rana et al. 2024a). Previous studies have indicated the presence of *Salmonella spp., Escherichia coli*, and *Staphylococcus aureus* in the beef value chain in Bangladesh, highlighting the requirement for excessive microbial surveillance in the food sector (Rana et al. 2024b). However, the occurrence and molecular detection of *K. pneumoniae* in this context remain underexplored in the beef value chain in Bangladesh. *K. pneumoniae* has the ability to form biofilms and is resistant to multiple antibiotics, which represents a rising threat within the food industry (Tanni et al. 2021).

Some food sources have the appearance of extended‐spectrum beta‐lactamases (ESBLs) and carbapenem‐resistant *K. pneumoniae* (CRKP) strains, which confound treatment possibilities and infection control measures (Husna et al. 2023; Effah et al. 2021). Studies found that *K. pneumoniae* can acquire resistance genes, which increases the risk of antimicrobial resistance propagation in both public health and animal populations (Kot and Witeska 2024). The simultaneous rise of antibiotic use in animal farming, particularly in growth‐promoting applications, has facilitated the presence of multidrug‐resistant *K. pneumoniae* in the dairy industry (Ferreira et al. 2019). The contamination of meat and meat products with MDR pathogens carries a serious public health threat, especially in urban markets where standards of hygiene practices vary significantly (Innes et al. 2021).

Despite the rising major global concerns regarding multi‐drug resistance (MDR), research on the comprehensive molecular epidemiology of *K. pneumoniae* in the beef value chain of Bangladesh remains limited. Some studies have mostly focused on the presence of MDR *Enterobacteriaceae* in dairy and poultry sectors, with limited focus on the role of raw beef and ready‐to‐eat beef products as a reservoir for MDR pathogens (Chowdhury et al. 2025; Hossain et al. 2008). The antibiotic resistance genes, genetic diversity, and virulence factors of *K. pneumoniae* strains in the beef value chain remain underexplored. Some previous studies suggest the potential of probiotic‐derived bacteriocins as an alternative antimicrobial tactic against MDR foodborne pathogens. A study highlighted the effectiveness of Lactobacillus acidophilus‐derived bacteriocins in combating MDR bacteria, including *K. pneumoniae*, indicating a promising intervention for improving food safety (Hasan et al. 2025). Similarly, a study characterized bacterial isolates from dairy sources and identified resistant strains, strengthening the need for heightened surveillance in the livestock industry (Ferdaus et al. 2019). Moreover, investigating the antimicrobial potential of probiotics provides a promising alternative approach for reducing the AMR risks associated with foodborne pathogens.

In a recent study of the beef value chain relevant to Bangladesh, it was found that infectious pathogens cause foodborne illnesses, significantly contributing to a considerable burden of disease and economic loss (Rana et al. 2024a). This study aimed to investigate the occurrence and antimicrobial resistance patterns of *K. pneumoniae* in the beef value chain, helping to outline policies for food safety and antimicrobial resistance. Additionally, findings will indicate the importance of strict hygiene practices across the beef value chain, encouraging improved regulations and surveillance mechanisms to protect against foodborne illnesses.

## 2 Materials and Methods

### 2.1 Sample Collection and Processing

**Figure 1:**
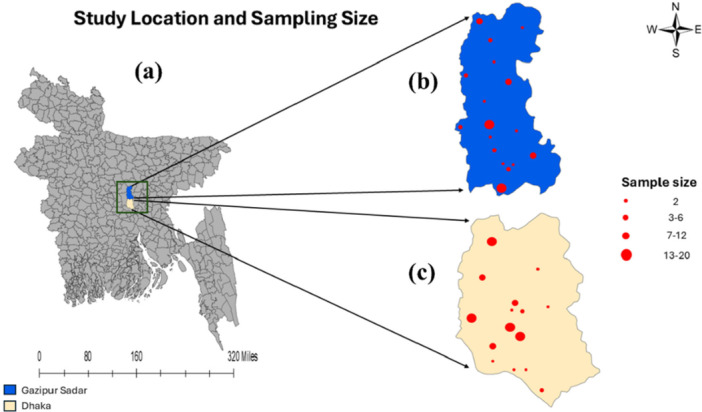
Sample collection and processing. Geo‐spatial mapping of the study area showing specific study location, sample size, and location‐based prevalence. (a) Geographical area of Bangladesh; Selected study area: (b) Gazipur City Corporation, and (c) Dhaka City Corporation.

A total of 240 samples were obtained from 6 categories comprising raw beef from slaughterhouse points, open butcher shops, and wet market selling points in mid‐afternoon and evening; Ready‐to‐eat (RTE) beef samples from high‐grade, medium‐grade, and poor‐grade restaurants (Supporting Information S1: Figure S1). This study was designed as a cross‐sectional study and was conducted between January and February 2023. Samples were collected aseptically from 30 places in the Dhaka City Corporation (DCC) and the Gazipur City Corporation (GCC) (Figure 1). Samples were kept in an ice box immediately after collection and transported to the Laboratory of Dairy Science and Laboratory of Pathology, Faculty of Veterinary, Animal & Biomedical Sciences, Sylhet Agricultural University.

All the samples were treated and cultured on Eosin Methylene Blue (HiMedia, India), and the microbial load was enumerated following the procedures according to our previous work (Roy et al. 2024). *K. pneumoniae* was identified based on colony morphology, and pure culture was prepared to perform antibiotic sensitivity test (AST) by Kirby‐Bauer disc diffusion method on Muller‐Hinton agar (HiMedia, India) plate described by (Hossain and Hossain 2019), following the guidelines of the Clinical and Laboratory Standards Institute (CLSI) 2020.

### 2.2 Molecular Detection of *K. pneumoniae* and Antimicrobial Resistance Genes (ARGs)

For molecular detection of *K. pneumoniae*, the universal gene was targeted following the procedures described by Al‐Arajy et al (Miller et al. 2013). with slight modifications. The oligonucleotide for universal 27 F 5′‐ GGTGTAGGATGAGACTATATA‐3’ and for universal 1392 R 5′‐TTCCATCTGCCTCTCCC‐3′ were used as the 16S rRNA gene primers for amplification and identification of *Klebsiella spp*. The PCR reaction was carried out in 25 µL reaction mixes that each contained 1 µL (10 p. mol/l) of the forward and reverse primers, 12.5 µL of the ready‐to‐use master mix (2x), 5.5 µL of nuclease‐free water, and finally, each reaction tube received a 5 µL addition of DNA template. The first stage involved denaturing the DNA (95°C for 5 min), and the second step included denaturing, annealing, and extension (35 cycles at 94°C for 30 s, 46°C for 30 s, and 72°C for 1 min 30 s), and the final extension step involved 72°C for 5 min. The PCR products were electrophoresed in 1.5% agarose gel and evaluated. The global 16S rRNA gene was confirmed to be positive for fragment sizes of around 1250 bp (Miller et al. 2013). A negative control was included in each PCR run to monitor contamination; however, a certified *K. pneumoniae* reference strain was not available for use as a positive control. Then, the partial sequencing (*16 s rRNA*) of *K. pneumoniae* was performed, and constructed the phylogenetic tree (Sequence submission no: PQ489347). ARGs were screened in the isolates by detection using PCR‐based amplification of all the targeted genes. All the primer sequences and product sizes were given in the table (Table S3).

### 2.3 Antimicrobial Susceptibility Testing

AST conducted by Kirby‐Bauer disc diffusion method on Muller‐Hinton agar plate described by (Hossain and Hossain 2019), following the guidelines of the Clinical and Laboratory Standards Institute (CLSI) 2020 (Supporting Information S1: Table S5). Quality control was ensured by strict adherence to CLSI‐recommended procedures, standardized inoculum preparation, and duplicate testing, although a CLSI‐recommended *K. pneumoniae* reference strain was not available for routine quality control. The susceptibility of *K. pneumoniae* isolates to 9 antibiotic class, which are commonly used on dairy and beef fattening farms, including Aminoglycosides: Streptomycin (S‐10 μg), Gentamycin (GEN‐10 μg), Amikacin (AK‐30 μg); Macrolides: Azithromycin (AZM‐15 μg); Tetracyclines: Tetracycline (TE‐30 μg); Quinolones: Ciprofloxacin (CIP‐5 μg), Norfloxacin (NOR‐10 μg); Sulfonamides: Sulfamethoxazole‐Trimethoprim (SXT‐25 μg); Penicillin: Ampicillin (AMP‐10 μg), Amoxicillin (AMX‐25 μg); Cephalosporins: Cefoxitin (CX‐30 μg), Ceftriaxone (CRO‐30 μg), Ceftazidime (CAZ‐30 μg); Carbapenem: Meropenem (MEM‐30 μg), and Imipenem (IPM‐30 μg).

### 2.4 Determination of Multiple Antibiotic Resistance Index (MARI)

The MARI for each isolate was calculated by the method described by Krumperman 1983 (Krumperman 1983) using the equation MAR index = *a*/*b*, where a represents the number of antibiotics to which the isolate was resistant, and *b* represents the total number of antibiotics against which the isolate was tested. A MARI > 0.2 indicates the existence of an isolate from high‐risk contaminated sources with frequent use of antibiotics, whereas value of 0.2 shows that bacteria are from sources that have been exposed to less antibiotic usage (Osundiya et al. 2013).

### 2.5 Detection of Biofilm Formation

#### 2.5.1 Congo Red Agar (CRA) Method

A simple qualitative method to detect biofilm formation by CRA was described by (Freeman et al. 1989) with slight modifications. CRA test was performed to detect the biofilm production by *K. pneumoniae* isolates recovered from raw beef and RTE samples. For this test, the medium was prepared by adding 0.8 g Congo red (HiMedia, India) to 1000 mL of blood agar, and fresh culture of *K. pneumoniae* was streaked on agar plates and incubated for 24–48 h at 37°C. Isolates showing black colonies with a dry crystalline appearance were considered strong biofilm producers. Weak biofilm producers were detected by a darkening of the colonies with the absence of a dry crystalline consistency, while non‐biofilm‐producing isolates showed smooth pink colonies on agar plates.

#### 2.5.2 Tissue Culture Plate Method

The tissue culture plate method was used to assess the biofilm‐forming ability of *K. pneumoniae* isolates with slight modification. Fresh cultures from agar plates were inoculated in 5 mL of trypticase soy broth (TSB) (Thermo Scientific Oxoid, United Kingdom) and were kept for incubation at 37°C for 24 h. A cell concentration of roughly 10^8^ CFU/mL was achieved for each isolate by adjusting the isolates' growth with a 0.5 McFarland concentration. After that, the cultures were serially diluted by a 10‐fold dilution method in fresh TSB, and 200 μL of the diluted culture was added to three wells of 96‐well flat‐bottomed microtiter polystyrene plates. Wells filled with TSB served as the negative controls. After the incubation at 37°C for 24 h, to eliminate planktonic bacteria or cells, thoroughly wash each microtiter plate 3 to 5 times using sterile PBS. The adhering cells were fixed with 95% ethanol for 5 min. Then, the plates were emptied, dried, and stained with 100 μL of 1% (V/V) crystal violet for a few minutes. After rinsing off excess stain with sterile distilled water, the plates were allowed to air dry. An automatic spectrophotometer (BioPremier, Portugal) was used to measure the optical density (OD) value at 570 nm (OD_570_ nm). Each isolate's biofilm formation assay yielded a score: strong biofilm producers (OD_570_ nm ≥ 1), moderate/intermediate biofilm producers (0.1 ≤ OD_570_ nm < 1), and non‐biofilm producers (OD_570_ nm < 0.1) (Kouidhi et al. 2010).

### 2.6 Phylogenetic Analysis and Comparison of *K. pneumoniae* From Different Sources and Countries in the Multilocus Sequence Typing Database

The ST phylogeny tree (Tamura 3‐parameter model) was constructed using the neighbor‐joining method in the MEGA11 software and was modified visually with the interactive tree of life (iTOL). The phylogenetic tree is tested by the bootstrap method, and the number of tests is 1000 times. The information on *K. pneumoniae* (taxid: 573) isolates from different countries and from different hosts was downloaded from the BLAST (NCBI) database (the same 100 STs as in this study). The minimum spanning tree (MST) was constructed by Bionumerics 8.1.

### 2.7 Statistical Analysis

All data obtained from the present study were entered into Microsoft Excel 2021 and analyzed. Graphical illustrations were produced using GraphPad Prism (version 10.3.1, San Diego, CA, USA) and RStudio (version 4.4.2, Boston, MA, USA). The prevalence of *Klebsiella pneumoniae* among different sample categories was compared using the Chi‐square (*χ*²) test. Microbial loads among sample types were evaluated using independent sample *t*‐tests or one‐way analysis of variance (ANOVA), as appropriate.

## 3 Results

### 3.1 Microbial Loads and Occurrence of *K. pneumoniae*

**Figure 2:**
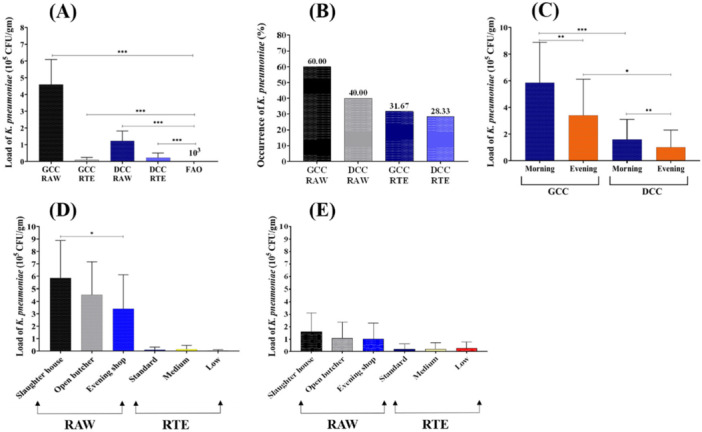
Microbial loads and occurrence of *Klebsiella pneumoniae*. Occurrence of *Klebsiella pneumoniae* in raw beef and ready‐to‐eat (RTE) samples. Samples of raw beef and RTE products were collected and processed to determine the (A) load of *K. pneumoniae*, (B) occurrence of *K. pneumoniae*, (C) load of *K. pneumoniae* over time, (D) load of *K. pneumoniae* in GCC, and (E) load of *K. pneumoniae* in DCC. The graphs illustrate the data for Gazipur City Corporation (GCC) and Dhaka City Corporation (DCC). The TAC values for three varieties of raw beef, RTEs, and the cumulative total, and comparison with the Standards recommended by the Food and Agricultural Organization (FAO). The dataset conveys the average ± SEM values derived from a sample size of 240. Statistical significance was determined using paired comparisons, where *p < 0.05, **p < 0.01, and ***p < 0.001.

We conducted an analysis on a total of 240 samples to assess the microbial load present in both raw beef and RTE samples. The Bangladesh Food Safety Authority (BFSA) has not established specific microbiological standards for *K. pneumoniae*. Also, the Food and Agriculture Organization (FAO) has not established standards, but it has established standards for the *Enterobacteriaceae* family (3 log 10 CFU/mL) (Kanko et al. 2023). *K. pneumoniae* is a Gram‐negative bacterium belonging to the family Enterobacteriaceae; therefore, FAO‐recommended Enterobacteriaceae limits were used as a reference for microbial safety assessment in this study. The results indicated that the bacterial load in both types of samples surpassed the safety threshold for microbial contamination as stipulated by the Food and Agriculture Organization (FAO) (Figure 2A and Supporting Information S1: Table S6). Among the samples, raw beef from GCC contained the highest number of *K. pneumoniae*, indicating the low level of hygienic practices during meat production. When comparing the values specified by FAO with those obtained from the analysis of the samples, it was observed that all categories of samples exceeded the standard limits for bacterial load (Figure 2B and Supporting Information S1: Table S6).

We collected samples of raw beer in the evening from the same open butcher shops, aiming to elucidate the temporal variations in *K. pneumoniae* bacterial loads over a period of 6–8 h in these raw beef samples. Remarkably, the evening raw beef samples exhibited bacterial populations that were 2 times higher than those observed in the morning samples (Figure 2C).

Of the total 240 samples examined, raw beef from GCC showed the highest occurrence of *K. pneumoniae*, with 60% of positive samples, while the lowest percentage of positive samples was in RTE from DCC, at 28.33%. 40% and 31.67% of samples tested positive in raw beef from DCC and RTE from GCC, respectively (Figure 2B and Supporting Information S1: Tables S1, S2). Remarkably, the raw beef samples from GCC exhibited bacterial loads that were 3–4 times higher than raw beef samples from DCC (Figure 2D,E).

### 3.2 Molecular Detection of *K. pneumoniae*

**Figure 3:**
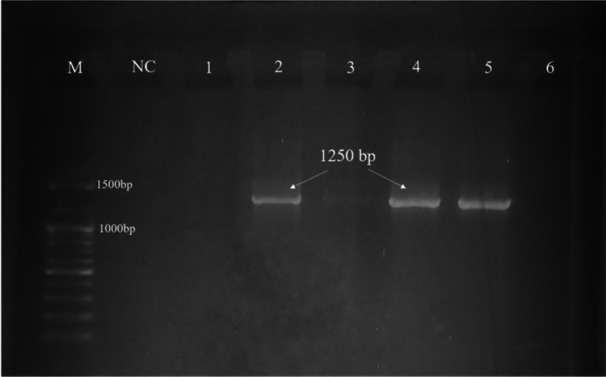
Molecular detection of *Klebsiella pneumoniae*. Molecular detection and 16 s rRNA sequencing of *K. pneumoniae* isolated from raw beef and RTE. Amplified DNA of *K. pneumoniae* isolates at 1250 bp. (Lane M: 100 bp DNA Ladder; Lane NC: (−ve) Control; Lane 2, 4, 5: *K. pneumoniae* positive isolates.

**Figure 4:**
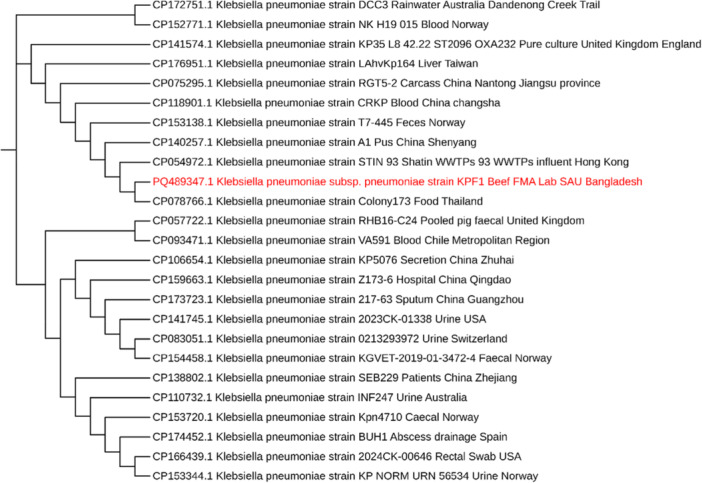
Phylogenetic tree. Phylogenetic tree based on the *16 s rRNA* gene sequence from this study and those from other reference strains, constructed by the neighbor‐joining method using MEGA software, version 11 (http://www.megasoftware.net/). The evolutionary history was inferred using the Neighbor‐Joining method. The optimal tree is shown (next to the branches). The evolutionary distances were computed using the Maximum Composite Likelihood method and are in the units of the number of base substitutions per site. All ambiguous positions were removed for each sequence pair (pairwise deletion option). GenBank accession no. PQ489347.

*K. pneumoniae* was confirmed by getting a specific band in the PCR result (Figure 3), consistent with previous studies that utilized universal 16 s rRNA primers for bacterial identification (Miller et al. 2013). The identification was more confirmed, followed by the partial sequencing of 16 s rRNA (Figure 4). The 16S rRNA gene‐based phylogenetic analysis placed the study isolate within the *K. pneumoniae* species cluster, where it grouped with reference sequences such as strain Colony173, isolated from food samples in Thailand (GenBank accession no. CP078766), and strain STIN93 Shatin WWTPs 93, isolated from wastewater influent in Hong Kong (GenBank accession no CP054972). This grouping reflects the conserved nature of the 16S rRNA gene and supports the taxonomic identification of the isolate as *K. pneumoniae*. Due to the limited discriminatory power of the 16S rRNA gene, this analysis does not provide resolution for strain‐level differentiation, transmission pathways, or epidemiological relationships.

### 3.3 Antibiogram Profile of *K. pneumoniae* Isolates

**Figure 5:**
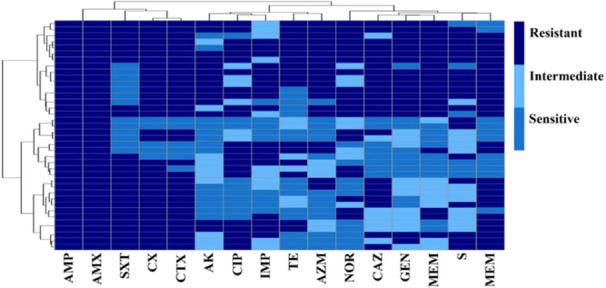
Antibiogram profile of *Klebsiella pneumoniae isolates*. Antibiogram profile of *Klebsiella pneumoniae* isolates. The Kirby‐Bauer disc diffusion method was employed for the antimicrobial susceptibility testing, revealing the resistance patterns of *K. pneumoniae* isolates obtained from raw beef and RTE samples from both GCC and DCC. The heat map visually represents the intensity of color from light blue to dark blue indicating sensitivity, intermediate sensitivity, and resistance. The tested antimicrobial agents include ampicillin (AMP), amoxicillin (AMX), sulfamethoxazole‐trimethoprim (SXT), cefoxitin (CX), cefotaxime (CTX), amikacin (AK), ciprofloxacin (CIP), imipenem (IMP), tetracycline (TE), azithromycin (AZM), norfloxacin (NOR), ceftazidime (CAZ), gentamycin (GEN), meropenem (MEM), and streptomycin (S).

The antimicrobial susceptibility test conducted using the disc diffusion method indicated that nearly all isolates of *K. pneumoniae* demonstrated resistance to most antibiotics recommended in the CLSI guidelines of 2020. Isolates of *K. pneumoniae* exhibited diverse patterns of antibiotic resistance. However, *K. pneumoniae* was completely resistant to ampicillin and amoxicillin (100%) (Figure 5 and Supporting Information S1: Table S7).

**Figure 6:**
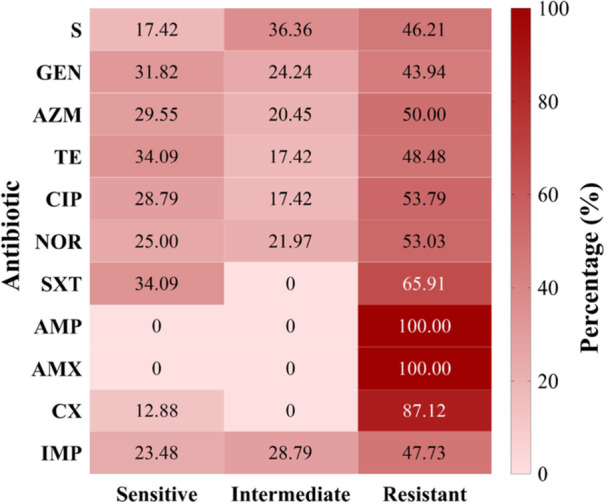
AMR profile of *Klebsiella pneumoniae isolates*. Antimicrobial resistance patterns of *Klebsiella pneumoniae* isolates. Antimicrobial susceptibility test by Kirby‐Bauer disc diffusion method. Heat map showing antimicrobial resistance patterns of the isolates of *K. pneumoniae* recovered from raw beef and RTE; All the isolates of *K. pneumoniae* were assessed for their antimicrobial susceptibility against CLSI‐2020 guided antimicrobial agents; The intensity of color from red to white in the vertical bar on the right side indicates the percentage of isolates from 0 to 100 respectively; The values inside each rectangle designate the percentage of resistance and sensitivity of isolates; S = Streptomycin, GEN = Gentamycin, AZM = Azithromycin, TE = Tetracycline, CIP = Ciprofloxacin, NOR = Norfloxacin, SXT = Sulfamethoxazole‐Trimethoprim, AMP = Ampicillin, AMX = Amoxicillin, CX = Cefoxitin.

Out of the 132 *K. pneumoniae* positive isolates, all exhibited complete resistance (100%) to ampicillin and amoxicillin as per the CLSI standards (Figure 6 and Supporting Information S1: Table S7). *K. pneumoniae* shows high resistance to cefoxitin (87.12%). In contrast, the highest sensitivity was observed with sulfamethoxazole‐trimethoprim and tetracycline (34.09%). Other antibiotics show a sensitivity ranging from 12.88% to 31.82%.

### 3.4 Multiple Antibiotic Resistance Index (MARI) of *K. Pneumoniae*

**Figure 7:**
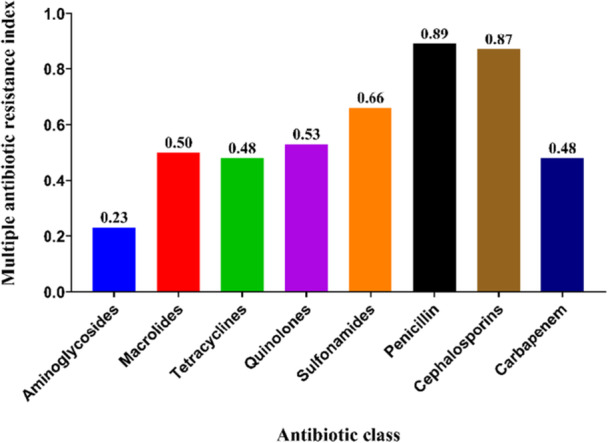
Multiple antibiotic resistance index (MARI) of *Klebsiella pneumoniae*. Multiple antibiotic resistance index (MARI) profiles of the *Klebsiella pneumoniae* isolates across different antimicrobial classes. All of the recovered *K. pneumoniae* were resistant to all antibiotic classes.

Figure 7 and Supporting Information S1: Table S8 show that all of the isolates had a MARI > 0.2. The highest MAR index shows the Penicillin and Cephalosporin group, at 0.89 and 0.87, respectively. Glycopeptides and aminoglycosides show the MAR index of 0.30 and 0.23, respectively. Other antibiotic classes show MARI between 0.48 and 0.66. The MAR index pattern observed was AMP^R^ –AMX^R^ – CX^R^ – SXT^R^ – CIP^R^ – NOR^R^ – LZD^R^ – AZM^R^ – TE^R^ – IMP^R^ – VA^R^ – S^R^ – GEN^R^.

### 3.5 ARGs

**Figure 8:**
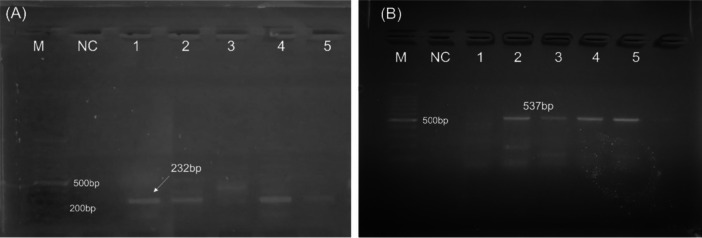
Antimicrobial resistant genes (ARGs). Molecular detection of antimicrobial resistant genes (ARGs) of *Klebsiella pneumoniae* isolates. Amplified DNA of ARGs of representative *K. pneumoniae* isolates. (A) Lane M, 100 bp DNA ladder; lane NC, control; lanes 1, 2, and 3, positive for *bla*_*IMP*_ at 232 bp. (B) Lane M, 100 bp DNA ladder; lane NC, control; lanes 2, 3, 4 and 5, positive for *bla*_*BIC*_ at 537 bp.

Overall, 132 positive isolates of *K. pneumoniae* from both raw beef and RTE samples were assayed for different resistance genes. Based on PCR results, environmental ARGs were found where the most prevalent ARGs was *bla*_*BIC*_ at 18.9%. The percentage of the *bla*_*IMP*_ gene was at 13.6%. Other ARGs were not found in our study (Figure 8 and Supporting Information S1: Table S4).

### 3.6 Biofilm Formation

**Figure 9:**
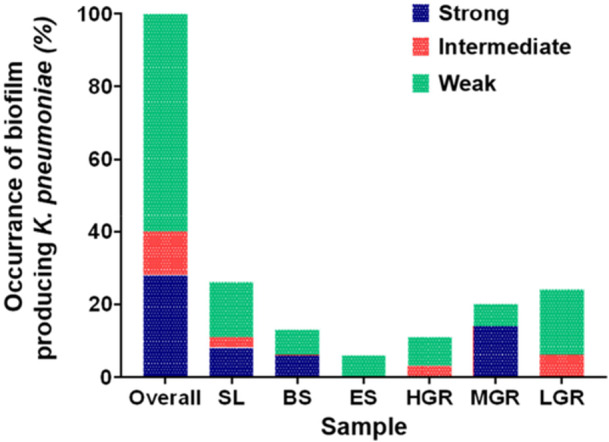
Biofilm formation of *Klebsiella pneumoniae*. Biofilm formation of *Klebsiella pnuemoniae*. The bar chart depicts the percentage of biofilm‐forming *K. pneumoniae* of 3 different categories (strong, intermediate, and weak biofilm producers). Samples were obtained from different types of raw beef markets and RTE sources. SL, slaughterhouse; BS, butcher shop; ES, evening shop; HGR, high‐grade restaurant; MGR, medium‐grade restaurant; LGR, low‐grade restaurant.

Isolates were categorized into strong, intermediate, and weak biofilm producers based on their characteristics observed on CRA plates (Supporting Information S1: Figure S2). Of the total isolates, 19% exhibited features indicative of biofilm production. Among these, 28% were classified as strong biofilm producers, while 12% were categorized as intermediate and 60% were weak biofilm producers (Figure 9 and Supporting Information S1: Table S9). Both CRA and tissue culture plate methods yielded comparable results, confirming the biofilm‐forming ability of a subset of *K. pneumoniae* isolates and minimizing methodological redundancy. The highest percentage of strong biofilm‐forming *K. pneumoniae* (14%) was found in medium‐grade RTE samples, while most of the isolates exhibited low producibility of biofilm.

## 4 Discussions

This discussion focuses on four key findings of the study: high contamination levels of *K. pneumoniae* in beef and RTE foods, widespread multidrug resistance, the presence of clinically relevant ARGs, and biofilm‐forming potential.

*K. pneumoniae* is an opportunistic pathogen that can colonize both humans and animals and is frequently found as a contaminant in retail meat (Multidrug‐Resistant Klebsiella pneumoniae Isolated from Farm Environments and Retail Products in Oklahoma ‐ ScienceDirect [Internet]). The discovery of antibiotic‐resistant *Klebsiella spp*. in food may enable public health professionals to develop methods that are more effective in minimizing contamination (Fahim et al. 2025). A thorough investigation was conducted to evaluate the contamination of *K. pneumoniae* in raw beef and RTE samples from the GCC and DCC of Bangladesh. The study highlights the load of *K. pneumoniae* and multidrug resistance and MAR index, emphasizing the urgent need for robust food safety measures to prevent foodborne illnesses associated with red meat consumption. These findings offer valuable insights for regulatory authorities and stakeholders, aiding the development of effective food safety protocols.

The study noted concerning levels of bacterial load of *K. pneumoniae* in raw beef and RTE samples from GCC and DCC, exceeding safety limits recommended by the FAO. Samples collected from the same shop in the morning and evening were assessed for bacterial load changes. Results demonstrated that the load of *K. pneumoniae* in morning samples was significantly higher than that of evening samples (Figure 2A–E). The presence of *K. pneumoniae* in raw beef and RTE samples poses significant health risks. Although cooking may reduce bacterial loads, finding pathogens in RTE samples emphasizes the need for strict food‐handling practices to prevent contamination and foodborne illnesses (Putri and Susanna 2021). The high bacterial loads observed in raw beef from GCC, compared to DCC, suggest differences in hygiene practices, storage conditions, and market infrastructure. Previous studies have indicated that unsanitary conditions in slaughterhouses and inadequate maintenance of cold chains contribute to the persistence of foodborne pathogens (Rana et al. 2024a).

The study indicated that *K. pneumoniae* occurrence in raw beef and RTE samples within the GCC and DCC regions. Our findings align with the previous study, analyzing 261 raw food samples, including meat, which reported a 43.7% contamination rate, with *K. pneumoniae* isolated from 44% of meat samples (Junaid et al. 2022). The samples analyzed in the Sylhet district of Bangladesh have shown that the overall prevalence of *Klebsiella spp*. was 28.89% (Liza et al. 2024). Research on minced meat in Egypt reported that 36% of packaged frozen and 88% of chilled unpackaged samples exceeded permissible microbial limits (El‐Kewaiey et al. 2015), while studies in Dhaka reported total aerobic bacterial counts in raw beef ranging from 6.2 to 6.5 log 10 CFU/g (Rahman 2021). Comparative research in northwest Spain demonstrated significant variation in microbial loads among establishments and methods of meat processing, underscoring the impact of hygiene standards on contamination levels (González‐Gutiérrez et al. 2019). These comparisons highlight that the contamination levels observed in GCC are consistent with finding from other low‐ and middle‐income settings where hygiene and cold‐chain maintenance remain suboptimal.

The high prevalence of *K. pneumoniae* in this study raises concerns about health risks from consuming contaminated beef and RTE products, particularly due to the presence of multidrug‐resistant isolates (Figures 5 and 6). AMR in foodborne bacteria is a significant global public health issue, and the high prevalence found in this study underscores the urgent need for antimicrobial stewardship and responsible antibiotic use in both human and veterinary medicine in Bangladesh (Islam et al. 2018). Among the antibiotics tested, all isolates exhibited complete resistance (100%) to ampicillin and amoxicillin, with high resistance to cefoxitin (87.12%). The highest levels of sensitivity were observed for sulfamethoxazole‐trimethoprim and tetracycline (34.09%), while other antibiotics showed limited effectiveness. Our findings align with a previous study indicating all isolates resistant to ampicillin, amoxicillin, cefuroxime, cefotaxime, and colistin; followed by the highest susceptibility observed towards gentamicin (97.95%), ciprofloxacin (85.71%), tetracycline (83.67%), and trimethoprim‐sulfamethoxazole (81.63%) (Liza et al. 2024). Another study found that 79% of isolates were resistant to cefuroxime, 77% to cefixime, and 65% to ceftriaxone (Tanni et al. 2021). The findings are closely aligned with the study (Ibrahim 2023), where ampicillin and cefuroxime show maximum resistance. In another study, it is depicted that gentamicin and ciprofloxacin are resistant to *K. pneumoniae* (Aminul et al. 2021), whereas gentamicin, ciprofloxacin, and trimethoprim‐sulfamethoxazole are moderately sensitive to *Klebsiella* portrayed by another study (Saha 2019). These discrepancies underscore the necessity for continuous local antibiotic susceptibility testing to inform effective treatment protocols.

*K. pneumoniae* is also a significant cause of nosocomial infections, with increasing antibiotic resistance. Studies across different regions show varying susceptibility profiles. In Nigeria, *K. pneumoniae* isolates from sputum samples demonstrated high susceptibility to levofloxacin, imipenem, ciprofloxacin, and gentamicin, but resistance to ampicillin and amoxicillin (Eyaufe and Okodua 2017). A Russian study found high resistance to multiple antibiotics, including carbapenems, with only fosfomycin and tigecycline showing low resistance rates (Santella et al. 2024). In India, *K*. *pneumoniae* from urinary tract infections showed the highest sensitivity to piperacillin‐tazobactam and amikacin, with 97.4% of isolates being multidrug‐resistant (Basavaraj and Jyothi 2014). A Nigerian study reported high susceptibility to imipenem, amikacin, cefoxitin, and aztreonam, but resistance to ampicillin and chloramphenicol (Romanus and Egwu 2011). These findings highlight the importance of local antibiotic susceptibility testing for effective treatment.

The multiple antibiotic resistance index (MARI), as shown in Figure 7 and Table S8, was an important analysis to check antibiotic resistance and health risk factors. All isolates exhibited a MAR index ≥ 0.2, indicating exposure to environments with frequent antibiotic use. The highest MAR index were observed for penicillin and cephalosporin groups, at 0.89 and 0.87, respectively, while other antibiotic classes showed MAR values ranging from 0.23 to 0.66. Similar MAR index values have been reported in Bangladesh and Nigeria, reflecting widespread antibiotic pressure in both clinical and food‐associated environments (Osundiya et al. 2013; Ogefere and Idoko 2024). Multiple studies have investigated antibiotic resistance in *K. pneumoniae*, revealing concerning trends. Research in Nigeria found high levels of multidrug resistance, with mean Multiple Antibiotic Resistance (MAR) indices ranging from 0.7994 to 0.82 for inpatients (Ogefere and Idoko 2024). Similar findings were reported in Lagos, with a MAR index of 0.4 for *K*. *pneumoniae* isolates (Osundiya et al. 2013).

In case of ARGs, the most prevalent ARGs were *bla*_*BIC*_ and *bla*_*IMP*_ at 18.9% and 13.6%, respectively (Figure 8 and Table S4). The complexity of antibiotic resistance in *K*. *pneumoniae* is further illustrated by a study identifying multiple beta‐lactamase genes in a single isolate, including TEM‐1, SHV variants, OXA‐9, and a plasmid‐mediated AmpC, with most resistance genes located on a large transferable plasmid (Hanson et al. 1999). These findings underscore the urgent need for antimicrobial stewardship and ongoing surveillance.

A crucial aspect of *K. pneumoniae* pathogenesis is biofilm formation, 19% exhibited features represents an additional challenge for food safety, as biofilms enhance persistence and resistance in processing and retail environments. This is consistent with findings from similar studies on foodborne pathogens in Bangladesh (Liu et al. 2023). While some studies have been conducted on chicken meat, fish, vegetables, and snacks in Bangladesh, there is a scarcity of data regarding similar studies on other food sources (ResearchGate [Internet]).

The increasing prevalence of AMR in *K. pneumoniae* within beef production systems presents a significant challenge to both animal and human health. Integrating a One Health approach that considers human, animal, and environmental interfaces will be essential to mitigate the spread of AMR and ensure sustainable beef production and public health protection.

### 4.1 Limitations

This study was confined to two urban settings and a limited sample size, which may not fully represent the spatial and seasonal diversity of the beef value chain across Bangladesh. Antimicrobial resistance was characterized using phenotypic susceptibility testing and PCR targeting selected resistance genes; however, the absence of whole‐genome sequencing (WGS) precluded comprehensive identification of the full resistome, virulome, plasmid content, and clonal relatedness of the isolates. Biofilm formation was assessed by in vitro phenotypic assays, which may not accurately reflect biofilm development and persistence under actual processing, storage, and retail conditions. Future studies incorporating WGS and broader geographic sampling would provide higher‐resolution insights into the transmission dynamics and public health risk of MDR *Klebsiella pneumoniae* along the beef value chain.

## 5 Conclusion

The findings of this study underscore the notable presence of *K. pneumoniae* in the beef value chain in Bangladesh, indicating its potential role as a reservoir for antimicrobial resistance (AMR). The elevated occurrence of *K. pneumoniae* in raw beef and ready‐to‐eat (RTE) meat samples, particularly in Gazipur City Corporation (GCC), suggests inadequate hygiene practices, inadequate maintenance of cold chains, and potential cross‐contamination during meat processing and distribution. The molecular detection and phylogenetic analysis of *K. pneumoniae* isolates revealed their genetic relatedness to strains implicated in foodborne infections in different countries, further emphasizing the potential risk of zoonotic transmission. Antimicrobial susceptibility testing revealed a troubling pattern of resistance, with all isolated strains exhibiting complete resistance to commonly used antibiotics, such as ampicillin and amoxicillin. Additionally, a significant level of resistance was observed against cefoxitin, while moderate susceptibility was detected for glycopeptides. These findings correspond with global trends in AMR, suggesting that the unregulated use of antibiotics in livestock production may be contributing to the selection and spread of MDR *K. pneumoniae* within the beef industry. Concerning the increasing public health threat posed by MDR *K. pneumoniae*, it is necessary to implement urgent interventions to mitigate its dissemination. Strengthening food safety regulations, enforcing stringent hygiene measures throughout the beef value chain, and implementing antimicrobial resistance programs are essential to reduce bacterial contamination and transmission of AMR. Additionally, further research is necessary to identify the genetic determinants of resistance and to develop effective surveillance systems for monitoring the emergence of resistant strains. Addressing these challenges is essential for ensuring food safety, protecting public health, and mitigating the burden of antimicrobial resistance in Bangladesh.

## Author Contributions

**Md Sodor Uddin:** writing – original draft, methodology, formal analysis, data curation. **Fahmida Jahan Fahim:** methodology, investigation, data curation. **Sohel Rana:** formal analysis, investigation, data curation. **Abdullah Al Kafi:** methodology. **Jobaida Khanam:** software, formal analysis. **Md Nazim Uddin:** validation, project administration. **Md Masudur Rahman:** project administration, conceptualization. **Monira Noor:** resources, project administration. **Md Mukter Hossain:** validation, conceptualization. **Md Mahfujur Rahman:** software, investigation, formal analysis. **Md Bashir Uddin:** validation, project administration. **Ferdaus Mohd Altaf Hossain:** writing – review and editing, supervision, conceptualization.

## Funding

The authors received no specific funding for this work.

## Ethics Statement

The authors have nothing to report.

## Conflicts of Interest

The authors declare no conflicts of interest.

## Supporting information


**Figure S1:** Samples collection sites. **Figure S2:** Characteristics of biofilm producing *Klebsiella pneumoniae* on CRA plates. **Table S1:** Prevalence of *Klebsiella pneumoniae* in raw beef and RTE samples at GCC. **Table S2:** Prevalence of *Klebsiella pneumoniae* in raw beef and RTE samples at DCC. **Table S3:** Primer sequences and product sizes. **Table S4:** Occurrence of *Klebsiella pneumoniae* and antibiotic‐resistant genes in raw and RTE samples based on PCR results. **Table S5:** Antibiotic resistance profile of *Klebsiella pneumoniae* isolated from raw red beef and RTE. **Table S6:** Microbial load of *Klebsiella pneumoniae* bacteria in raw and RTE samples. **Table S7:** Antimicrobial resistance patterns (Percentage of *Klebsiella pneumoniae* isolates). **Table S8:** Multiple antibiotic resistance index (MARI) profile of the *Klebsiella pneumoniae* isolates. **Table S9:** Occurrence of biofilm formation in raw and ready‐to‐eat (RTE) samples.

## Data Availability

The data supporting the findings of this study are available upon request to the corresponding author. Requests for access to the data should be addressed to corresponding author. Please include a brief description of the purpose for which the data is requested, and approval will be granted in accordance with any applicable data usage restrictions.
