# Supplementary material for: Emerging Antimicrobial Resistance in Klebsiella pneumoniae: A Molecular and Antibiogram Insight From the Beef Value Chain in Bangladesh
Source: Microbiologyopen. 2026 Mar 25;15(2):e70263. doi: 10.1002/mbo3.70263 (PMC13140612; doi:10.1002/mbo3.70263)
Supplement: Supplementary file 1 — Figure S1: Samples collection sites. Figure S2: Characteristics of biofilm producing Klebsiella pneumoniae on CRA plates. Table S1: Prevalence of Klebsiella pneumoniae in raw beef and RTE samples at GCC. Table S2: Prevalence of Klebsiella pneumoniae in raw beef and RTE samples at DCC. Table S3: Primer sequences and product sizes. Table S4: Occurrence of Klebsiella pneumoniae and antibiotic‐resistant genes in raw and RTE samples based on PCR results. Table S5: Antibiotic resistance profile of Klebsiella pneumoniae isolated from raw red beef and RTE. Table S6: Microbial load of Klebsiella pneumoniae bacteria in raw and RTE samples. Table S7: Antimicrobial resistance patterns (Percentage of Klebsiella pneumoniae isolates). Table S8: Multiple antibiotic resistance index (MARI) profile of the Klebsiella pneumoniae isolates. Table S9: Occurrence of biofilm formation in raw and ready‐to‐eat (RTE) samples. [file MBO3-15-e70263-s001.docx]

**Supplementary Figures**


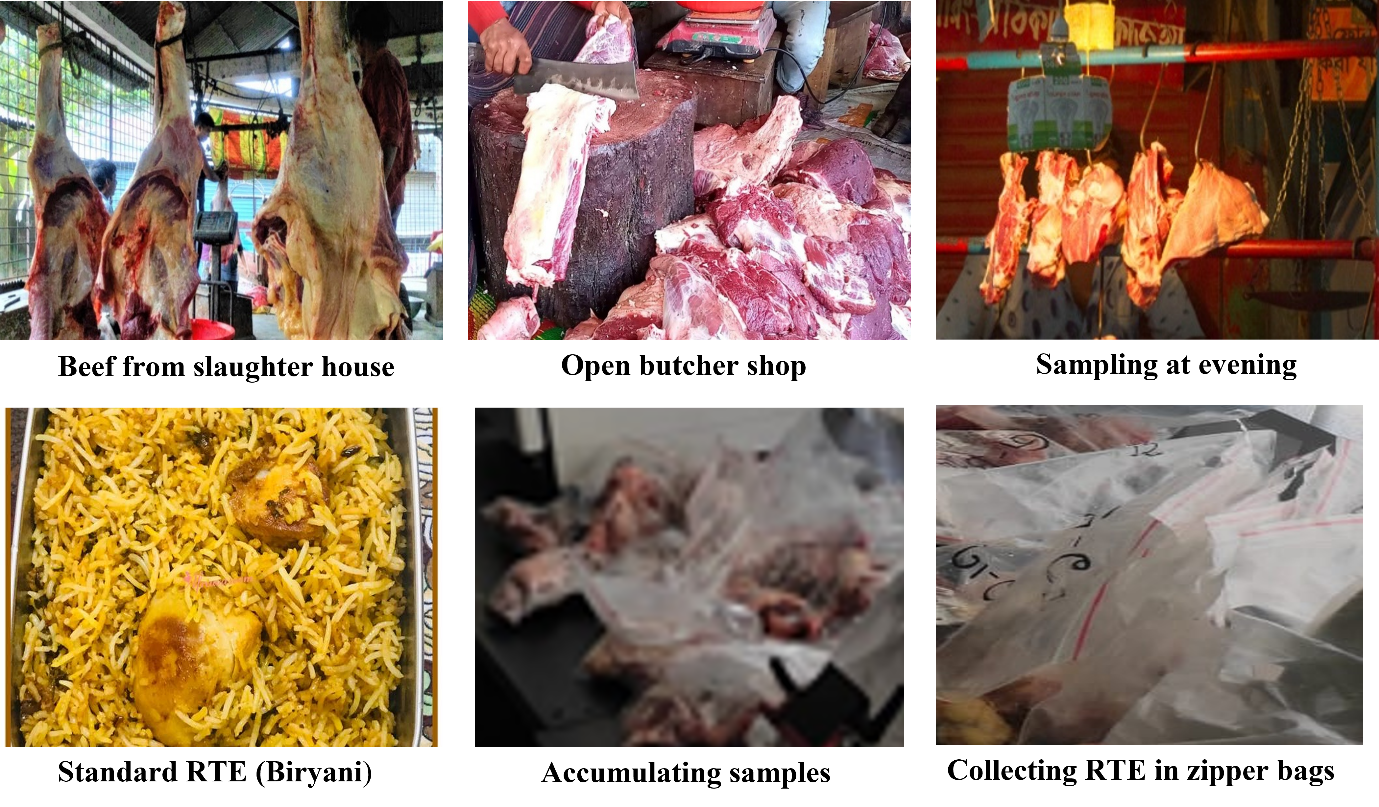


**Figure S1: Samples collection sites**

**
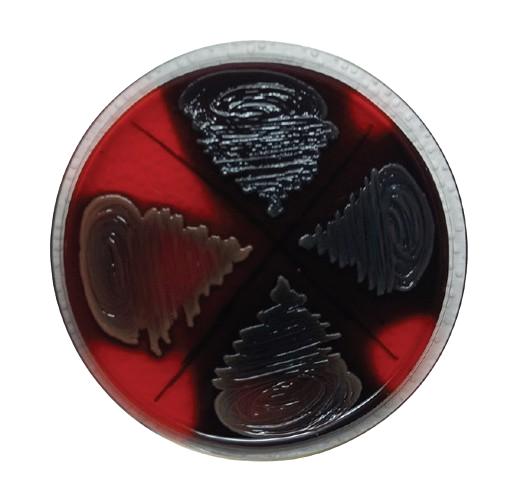
**

**Figure S2:** Characteristics of biofilm producing *Klebsiella pneumoniae* on CRA plates.

**Supplementary Tables**

**Table S1: Prevalence of *Klebsiella pneumoniae* in raw beef and RTE samples at GCC**.

| **GCC RAW (n=60)** | | | | **GCC RTE (n=60)** | | | |
| --- | --- | --- | --- | --- | --- | --- | --- |
| **Slaughter house (n=20)** | **Open butcher (n=20)** | **Evening shop (n=20)** | **Total Raw (n=60)** | **Standard (n=20)** | **Medium (n=20)** | **Low (n=20)** | **Total RTE (n=60)** |
| 20 (100%) | 20 (100%) | 20 (100%) | 60 (100%) | 4 (20%) | 9 (45%) | 6 (30%) | 19 (31.67%) |

Abbreviation: GCC, Gazipur City Corporation; RTE, Ready-to-Eat

**Table S2: Prevalence of *Klebsiella pneumoniae* in raw beef and RTE samples at DCC**.

| **DCC RAW (n=60)** | | | | **DCC RTE (n=60)** | | | |
| --- | --- | --- | --- | --- | --- | --- | --- |
| **Slaughter house (n=20)** | **Open butcher (n=20)** | **Evening shop (n=20)** | **Total Raw (n=60)** | **Standard (n=20)** | **Medium (n=20)** | **Low (n=20)** | **Total RTE (n=60)** |
| 8 (40%) | 15 (75%) | 13 (65%) | 36 (60%) | 7 (35%) | 4 (20%) | 6 (30%) | 17 (28.33%) |

Abbreviation: DCC, Dhaka City Corporation; RTE, Ready-to-Eat

**Table S3: Primer sequences and product sizes**

| **Target gene** | **Primer Sequences (5’-3’)** | **Size of Product (bp)** |
| --- | --- | --- |
| *mcr-1* | AGTCCGTTTGTTCTTGTGGC  AGATCCTTGGTCTCGGCTTG | 320 |
| *mcr-2* | CAAGTGTGTTGGTCGCAGTT  TCTAGCCCGACAAGCATACC | 715 |
| *mcr-3* | AAATAAAAATTGTTCCGCTTATG  AATGGAGATCCCCGTTTTT | 929 |
| *mcr-4* | TCACTTTCATCACTGCGTTG  TTGGTCCATGACTACCAATG | 1116 |
| *mcr-5* | ATGCGGTTGTCTGCATTTATC  TCATTGTGGTTGTCCTTTTCTG | 1644 |
| *mcr-6* | AGCTATGTCAATCCCGTGAT  ATTGGCTAGGTTGTCAATC | 252 |
| *mcr-7* | GCCCTTCTTTTCGTTGTT  GGTTGGTCTCTTTCTCGT | 551 |
| *mcr-8* | TCAACAATTCTACAAAGCGTG  AATGCTGCGCGAATGAAG | 856 |
| *mcr-9* | TTCCCTTTGTTCTGGTTG  GCAGGTAATAAGTCGGTC | 1011 |
| *vanA* | GTAGGCTGCGATATTCAAAGC  CGATTCAATTGCGTAGTCCAA | 732 |
| *vanB* | GGTATCAAGGAAACCTC  CTTCCGCCATCATAGCT | 625 |
| *bla_IMP_* | GGAATAGAGTGGCTTAACTCTC  CGAATGCGCACCAG | 232 |
| *bla_VIM_* | TGGTGTTTGGTCGCAAT  CGAATGCGCACCAG | 390 |
| *bla_NDM_* | GGTTTGGCGATCTGGTTTTC  CGGAATGGCTCATCACGATC | 621 |
| *bla_KPC_* | CGTCTAGTTCTGCTGTCTTG  CTTGTCATCCTTGTTAGGCG | 798 |
| *bla_BIC_* | TATGCAGCTCCTTTAAGGGC  TCATTGGCGGTGCCGTACAC | 537 |
| *bla_OXA-48_* | GCGTGGTTAAGGATGAACAC  CATCAAGTTCAACCCAACCG | 438 |

**Table S4: Occurrence of *Klebsiella pneumoniae* and antibiotic-resistant genes in raw and RTE samples based on PCR results**

| **Target gene** | **No. of positive isolates/Total isolates** | **Occurrence (%)** |
| --- | --- | --- |
| 16s rRNA | 132/240 | 55 |
| *mcr-1* | 0/132 | 0 |
| *mcr-2* | 0/132 | 0 |
| *mcr-3* | 0/132 | 0 |
| *mcr-4* | 0/132 | 0 |
| *mcr-5* | 0/132 | 0 |
| *mcr-6* | 0/132 | 0 |
| *mcr-7* | 0/132 | 0 |
| *mcr-8* | 0/132 | 0 |
| *mcr-9* | 0/132 | 0 |
| *vanA* | 0/132 | 0 |
| *vanB* | 0/132 | 0 |
| *bla_IMP_* | 18/132 | 13.6 |
| *bla_VIM_* | 0/132 | 0 |
| *bla_NDM_* | 0/132 | 0 |
| *bla_KPC_* | 0/132 | 0 |
| *bla_BIC_* | 25/132 | 18.9 |
| *bla_OXA-48_* | 0/132 | 0 |

**Table S5: Antibiotic resistance profile of *Klebsiella pneumoniae* isolated from raw red beef and RTE**

| **Antibiotic class** | **Specific antibiotic tested** | **Concentration** | **Interpretive categories and zone diameter breakpoints (nearest whole mm)** | | | **No. of isolates/Total isolates** | | | |
| --- | --- | --- | --- | --- | --- | --- | --- | --- | --- |
|  |  |  | **S** | **I** | **R** | | **S** | **I** | **R** |
| Aminoglycosides | Streptomycin | 10 μg | ≥ 15 | 12-14 | ≤ 11 | | 6/38 | 12/38 | 20/38 |
|  | Gentamycin | 10 μg | ≥ 15 | 13-14 | ≤ 12 | | 10/38 | 11/38 | 17/38 |
|  | Amikacin | 30 μg | ≥ 17 | 15-16 | ≤ 14 | | 14/38 | 10/38 | 14/38 |
| Macrolides | Azithromycin | 15 μg | ≥ 13 | - | ≤ 12 | | 10/38 | 8/38 | 20/38 |
| Tetracyclines | Tetracycline | 30 μg | ≥ 15 | 12-14 | ≤ 11 | | 15/38 | 6/38 | 17/38 |
| Quinolones | Ciprofloxacin | 5 μg | ≥ 26 | 22-25 | ≤ 21 | | 10/38 | 6/38 | 22/38 |
|  | Norfloxacin | 10 μg | ≥ 17 | 13-16 | ≤ 12 | | 10/38 | 8/38 | 20/38 |
| Sulfonamides | Sulfamethoxazole-Trimethoprim | 25 μg | ≥ 16 | 11-15 | ≤ 10 | | 13/38 | 0/38 | 25/38 |
| Penicillin | Ampicillin | 10 μg | ≥ 17 | 14-16 | ≤13 | | 0/38 | 0/38 | 38/38 |
|  | Amoxicillin | 25 μg | ≥ 18 | 14-17 | ≤ 13 | | 0/38 | 0/38 | 38/38 |
| Cephalosporins | Cefoxitin | 30 μg | ≥ 18 | 15-17 | ≤ 14 | | 5/38 | 0/38 | 33/38 |
|  | Ceftriaxone | 30 μg | ≥ 23 | 20-22 | ≤19 | | 6/38 | 0/38 | 32/38 |
|  | Ceftazidime | 30 μg | ≥ 21 | 18-20 | ≤ 17 | | 8/38 | 7/38 | 23/38 |
| Monobactam | Aztreonam | 30 μg | ≥ 21 | 18-20 | ≤ 17 | | 14/38 | 5/38 | 19/38 |
| Carbapenem | Meropenem | 30 μg | ≥ 23 | 20-22 | ≤ 19 | | 11/38 | 0/38 | 27/38 |
|  | Imipenem | 30 μg | ≥ 23 | 20-22 | ≤ 19 | | 9/38 | 11/38 | 18/38 |

Abbreviation: S, sensitive; I, intermediate; R, resistant; mm, millimeter

**Table S6: Microbial load of *Klebsiella pneumoniae* bacteria in raw and RTE samples**

| GCC-RAW | CFU/gm (10^5^) | GCC-RTE | CFU/gm (10^5^) | DCC-RAW | CFU/gm (10^5^) | DCC-RTE | CFU/gm (10^5^) |
| --- | --- | --- | --- | --- | --- | --- | --- |
| A1 | 6.3 | H1 | 0 | A1 | 3.65 | H1 | 0 |
| A2 | 7.4 | H2 | 0.44 | A2 | 3.52 | H2 | 0 |
| A3 | 5.2 | H3 | 0 | A3 | 3.2 | H3 | 0 |
| A4 | 3.27 | H4 | 0 | A4 | 0 | H4 | 0 |
| A5 | 6.25 | H5 | 0 | A5 | 3.1 | H5 | 0.22 |
| A6 | 4.65 | H6 | 0 | A6 | 2.5 | H6 | 0 |
| A7 | 3.65 | H7 | 0.45 | A7 | 3.6 | H7 | 0 |
| A8 | 3.87 | H8 | 0 | A8 | 0.93 | H8 | 0 |
| A9 | 5 | H9 | 0 | A9 | 0 | H9 | 0.1 |
| A10 | 4.61 | H10 | 0 | A10 | 3.2 | H10 | 0 |
| A11 | 3.92 | H11 | 0 | A11 | 3.12 | H11 | 0.5 |
| A12 | 13 | H12 | 0 | A12 | 2.02 | H12 | 0 |
| A13 | 3.86 | H13 | 0 | A13 | 0 | H13 | 0.65 |
| A14 | 3.49 | H14 | 0.5 | A14 | 1.4 | H14 | 0.12 |
| A15 | 12 | H15 | 0.65 | A15 | 0 | H15 | 0 |
| A16 | 10.2 | H16 | 0 | A16 | 0 | H16 | 0 |
| A17 | 4.12 | H17 | 0 | A17 | 0.24 | H17 | 1.2 |
| A18 | 2.98 | H18 | 0 | A18 | 0 | H18 | 1.4 |
| A19 | 3.63 | H19 | 0 | A19 | 1.5 | H19 | 0 |
| A20 | 9.7 | H20 | 0 | A20 | 0 | H20 | 0 |
| B1 | 6.9 | M1 | 0.71 | B1 | 0.38 | M1 | 1.32 |
| B2 | 7.9 | M2 | 0.05 | B2 | 0.27 | M2 | 0 |
| B3 | 3.45 | M3 | 0.01 | B3 | 0 | M3 | 0.56 |
| B4 | 2.22 | M4 | 0.01 | B4 | 0.66 | M4 | 0 |
| B5 | 5.67 | M5 | 0.05 | B5 | 0.35 | M5 | 0 |
| B6 | 8.9 | M6 | 0 | B6 | 3.2 | M6 | 0 |
| B7 | 1.93 | M7 | 0 | B7 | 0.47 | M7 | 0 |
| B8 | 3.89 | M8 | 0 | B8 | 3.4 | M8 | 1.8 |
| B9 | 9.1 | M9 | 0.12 | B9 | 3.6 | M9 | 0 |
| B10 | 3.25 | M10 | 0 | B10 | 0.32 | M10 | 0 |
| B11 | 2.31 | M11 | 0 | B11 | 2.5 | M11 | 0 |
| B12 | 1.65 | M12 | 0 | B12 | 0.26 | M12 | 0 |
| B13 | 3.59 | M13 | 0.05 | B13 | 0.37 | M13 | 0 |
| B14 | 4.8 | M14 | 1.12 | B14 | 0 | M14 | 0 |
| B15 | 2.55 | M15 | 0 | B15 | 1.9 | M15 | 0 |
| B16 | 7.6 | M16 | 0 | B16 | 2.4 | M16 | 0 |
| B17 | 2.65 | M17 | 0 | B17 | 1.6 | M17 | 0.4 |
| B18 | 1.36 | M18 | 0 | B18 | 0 | M18 | 0 |
| B19 | 8.2 | M19 | 0.68 | B19 | 0 | M19 | 0 |
| B20 | 2.89 | M20 | 0 | B20 | 0 | M20 | 0 |
| C1 | 2.82 | L1 | 0 | C1 | 0 | L1 | 1.6 |
| C2 | 1.88 | L2 | 0 | C2 | 0 | L2 | 0.21 |
| C3 | 3.13 | L3 | 0 | C3 | 3.1 | L3 | 0 |
| C4 | 1.63 | L4 | 0.07 | C4 | 0 | L4 | 0.56 |
| C5 | 1.96 | L5 | 0.02 | C5 | 0 | L5 | 0 |
| C6 | 2.35 | L6 | 0 | C6 | 0 | L6 | 0 |
| C7 | 8.2 | L7 | 0.1 | C7 | 2.41 | L7 | 0 |
| C8 | 0.87 | L8 | 0 | C8 | 0 | L8 | 0 |
| C9 | 4.5 | L9 | 0 | C9 | 0 | L9 | 0 |
| C10 | 1.43 | L10 | 0 | C10 | 0 | L10 | 1.3 |
| C11 | 1.56 | L11 | 0 | C11 | 0 | L11 | 0 |
| C12 | 1.93 | L12 | 0 | C12 | 2.5 | L12 | 0 |
| C13 | 9.4 | L13 | 0.15 | C13 | 0 | L13 | 0 |
| C14 | 1.65 | L14 | 0.23 | C14 | 0 | L14 | 0 |
| C15 | 1.85 | L15 | 0 | C15 | 0 | L15 | 0 |
| C16 | 7.64 | L16 | 0 | C16 | 2.1 | L16 | 1.2 |
| C17 | 1.95 | L17 | 0 | C17 | 3.02 | L17 | 0 |
| C18 | 8.56 | L18 | 0.14 | C18 | 2.6 | L18 | 0.5 |
| C19 | 2.1 | L19 | 0 | C19 | 2.4 | L19 | 0 |
| C20 | 2.65 | L20 | 0 | C20 | 2.1 | L20 | 0 |

**Table S7: Antimicrobial resistance patterns (Percentage of *Klebsiella pneumoniae* isolates)**

| **Antibiotic class** | **Antibiotics** | **Percentage of the isolates (%)** | | |
| --- | --- | --- | --- | --- |
|  |  | **Resistant** | **Intermediate** | **Sensitive** |
| Aminoglycosides | Streptomycin | 46.21 | 36.36 | 17.42 |
|  | Gentamycin | 43.94 | 24.24 | 31.82 |
| Macrolides | Azithromycin | 50.00 | 20.45 | 29.55 |
| Tetracyclines | Tetracycline | 48.48 | 17.42 | 34.09 |
| Quinolones | Ciprofloxacin | 53.79 | 17.42 | 28.79 |
|  | Norfloxacin | 53.03 | 21.97 | 25.00 |
| Sulfonamides | Sulfamethoxazole-Trimethoprim | 65.91 | 0.00 | 34.09 |
| Penicillin | Ampicillin | 100.00 | 0.00 | 0.00 |
|  | Amoxicillin | 100.00 | 0.00 | 0.00 |
| Cephalosporins | Cefoxitin | 87.12 | 0.00 | 12.88 |
| Carbapenem | Imipenem | 47.73 | 28.79 | 23.48 |

**Table S8: Multiple antibiotic resistance index (MARI) profile of the *Klebsiella pneumoniae* isolates**

| Antibiotic class | No of antibiotics | Resistance phenotypes | No. of *K. pneomoniae isolates (n = 132)* | MARI |
| --- | --- | --- | --- | --- |
| Aminoglycosides | 2 | S^R^, GEN^R^ | 59.5 (45.1%) | 0.23 |
| Macrolides | 1 | AZM^R^ | 66 (50%) | 0.50 |
| Tetracyclines | 1 | TE^R^ | 64 (48.5%) | 0.48 |
| Quinolones | 2 | CIP^R^, NOR^R^ | 70.5 (53.4%) | 0.53 |
| Sulfonamides | 1 | SXT^R^ | 87 (65.9%) | 0.66 |
| Penicillin | 3 | AMP^R^, AMX^R^ | 118 (89.4%) | 0.89 |
| Cephalosporins | 1 | CX^R^ | 115 (87.1%) | 0.87 |
| Carbapenem | 1 | IMP^R^ | 63 (47.7%) | 0.48 |

Abbreviation: S-Streptomycin; GEN-Gentamycin; AZM-Azithromycin; TE-Tetracycline; CIP-Ciprofloxacin; NOR-Norfloxacin; SXT-Sulfamethoxazole Trimethoprim; AMP-Ampicillin; AMX-Amoxicillin; CX-Cefoxitin

**Table S9: Occurrence of biofilm formation in raw and ready-to-eat (RTE) samples**

| **Biofilm formation** | **No. of positive isolates/Total isolates** | **Occurrence (%)** |
| --- | --- | --- |
| Biofilm producer | 25/132 | 19 |
| Strong | 7 | 28 |
| Intermediate | 3 | 12 |
| Weak | 15 | 60 |
